# Teaching residents to put patients first: creation and evaluation of a comprehensive curriculum in patient-centered communication

**DOI:** 10.1186/s12909-018-1371-3

**Published:** 2018-11-19

**Authors:** Dorothea Wild, Haq Nawaz, Saif Ullah, Christina Via, William Vance, Paul Petraro

**Affiliations:** 1 Preventive Medicine Residency Program, Griffin Hospital, Derby, USA; 2 Combined Internal Medicine/Preventive Medicine Residency Program, Griffin Hospital, Derby, United States; 3 Department of Medical Education, Griffin Hospital, 130 Division Street, Derby, CT 06484 USA; 4 Yale University, New Haven, USA

**Keywords:** Curriculum, Resident, Curriculum evaluation, Physician-patient communication, Patient-centered communication

## Abstract

**Background:**

Patient-centered communication is essential for successful patient encounters and positive patient outcomes. Therefore, training residents how to communicate well is one of the key responsibilities of residency programs. However, many residents, especially international medical graduates, continue to struggle with communication barriers.

**Methods:**

All residents and faculty from a small community teaching hospital participated in a three-year, multidimensional patient-centered communication curriculum including communication training with lectures, experiential learning, communication skills practice, and reflection in the areas of linguistics, physician-patient communication, cultural & linguistically appropriate care, and professionalism. We evaluated the program through a multipronged outcomes assessment, including self-assessment, scores on the Calgary-Cambridge Scale during Objective Structured Clinical Examination (OSCE), a survey to measure the hidden curriculum, English Communication Assessment Profile (E-CAP),, the Maslach Burnout-Inventory (MBI), and residents’ evaluation of faculty communication.

**Results:**

Sixty-two residents and ten faculty members completed the three-year curriculum. We saw no significant changes in the MBI or hidden curriculum survey. Communication skills as measured by Calgary Cambridge Score, E-CAP, and resident communication improved significantly (average Calgary-Cambridge Scale scores from 70% at baseline to 78% at follow-up (*p*-value < 0.001), paired t-test score from 68% at baseline to 81% at follow-up (p-value < 0.004), average E-CAP score from 73 to 77% (p-value < 0.001)). Faculty communication and teaching as rated by residents also showed significant improvement in four out of six domains (learning climate (*p* < 0.001), patient-centered care (*p* = 0.01), evaluation (*p* = 0.03), and self-directed learning (p = 0.03)).

**Conclusion:**

Implementing a multidimensional curriculum in patient-centered communication led to modest improvements in patient-centered communication, improved language skills, and improved communication skills among residents and faculty.

**Electronic supplementary material:**

The online version of this article (10.1186/s12909-018-1371-3) contains supplementary material, which is available to authorized users.

## Background

The practice of medicine requires good communication skills to foster excellent rapport in the doctor-patient relationship [1–3]. Communication is one of the six core competencies defined by the Accreditation Council for Graduate Medical Education (ACGME). Beyond fostering a good relationship, empathetic communication skills that allow patients to express their views are also associated with improved patient outcomes [2, 3]. In addition to the direct impact on patient outcomes, improved physician communication may also lead to better inter-professional communication, which in turn is associated with better patient safety [4]. The residency period is uniquely suitable for longitudinal, extended communication training in which skills can be developed and integrated [1–3]. Despite the critical nature of these competencies in practice, the teaching of communication skills to medical students and residents is often informal through an apprenticeship model without formal feedback [5]. Several multimodal curricula to improve residents’ communication through skills practice, lectures, and taped or supervised encounters have been tested previously and led to increased use of recommended communication behavior [6, 7] and trainee self-efficacy [8]. However, these curricula only address residents’ direct communication skills.

Beyond direct interactions with teaching faculty, residents’ communication is also affected by socio-demographic factors, cultural norms, and organization culture. For example, organizational norms around what constitutes acceptable behavior determine substantially which behaviors residents adopt permanently and what they consider acceptable [2, 3]. Such organizational norms for expected behavior have been called the “hidden curriculum” and been shown to influence actual trainee behavior with patients much more deeply than explicit values or curricula [9].

### International medical graduates

Some residents and particularly international medical graduates struggle with communicating clearly because of foreign accent, suboptimal information sequencing, and stilted language [10]. In addition, medical professionalism as a cultural concept may well vary with location and cultural context [11]. A previous study of communication training for foreign medical graduates included self-reflection and reported improved confidence in communication [12]. However, no formal assessment or remediation for particular communication challenges were described.

Therefore, sustained improvement in residents’ communication skills may be achieved by integrating linguistic/communication training with other communication training in the setting of adequate role modeling by teaching faculty [13]. Furthermore, the evaluation of residents’ communication should be guided by direct observation, similar to other competencies and procedural skills [14]. To our knowledge, a similarly comprehensive curriculum has so far not been implemented and evaluated. We therefore designed and evaluated a multimodal, comprehensive curriculum of patient-centered communication targeting both residents and their faculty preceptors.

## Methods

Our aim was to implement a multimodal patient-centered communication curriculum and to assess the impact of this curriculum on residents’ professionalism, communication skills, and the hidden curriculum of the organization through a before/after design. We incorporated a broad range of approaches to improve face-to-face communication skills: linguistic, physician-patient communication, culturally and linguistically appropriate care, and introduced direct observation at multiple points of the curriculum.

### Setting and participants

Griffin Hospital is a 140-bed community teaching hospital in southern Connecticut, which trains residents in an internal medicine program (12 residents), a preliminary year program (9 residents), and a combined internal medicine/preventive medicine program (12 residents). The teaching program admits approximately 4000 patients/year, of which approximately 12.5% are Medicaid, self-pay, or uninsured. During the time of the program, 95% of the admissions to medicine were to the teaching service and trained for by residents. At the time, the program had 10 attending physicians providing the bulk of the teaching interactions (four program directors/associated program directors, one geriatrician, five hospitalists).

### Program description

Beginning in 2010, we started to implement our communication curriculum with the help of Primary Care Residency Training grant from HRSA (2010–2012). This curriculum included educational activities focusing on clear speaking, content and structure of patient-centered communication, culturally and linguistically appropriate care, and professionalism, supplemented by efforts to train the main attending physicians. Table 1 shows an overview of the curricular content, methods, time spent, and evaluation methods. We taught each educational domain through a combination of curricular activities. These included lectures, experiential components (direct observation of patient interactions, skills exercises or review of videotaped patient encounters), reflections and (for patient-centered communication) workshops. Prior to this curriculum, our program offered daily one-hour noon lectures and weekly grand rounds on various medical topics. For the duration of the study, we replaced one lecture/week and one grand round/month with communication content and added the patient-centered communication workshops. Grant funding was used to support monthly faculty development sessions, the patient-centered communication program, some outside lectures, developing the OSCE scenarios, and the program evaluation. The health literacy rotation was newly created and required about 2 h of faculty time/rotating resident and week. The other teaching activities as well as the communication OSCEs were integrated into the existing residency program activities by streamlining educational activities; this did not require extra time. For example, reviewing the OSCE tapes became part of previously existing biannual meetings between program director and residents. All residents participated in the health literacy rotation and the comprehensive baseline skills assessment. For participation in the other activities, we estimate that the participation rate was around 75% for the noon conferences and linguistic 3 h sessions.

**Table 1:** Curricular Activity and Evaluation Method

Domain	Perspective on communication	Curricular activity	Time investment	Taught by	Evaluation
Clear speaking	Linguistic	Communication clarity training by linguist (web-based individual assessment, lectures; home exercises with individual feedback)	3 h-sessions every 3 months	Specialized linguist	Before and after E-CAP®
Content and Strategy of Patient-Centered Communication	Physician-patient communication	Lectures, review of videotapes of resident-patient interactions Integration of communication training in work-rounds	1.5 h interactive seminar every month	Expert Faculty	OSCE: at least 2/year HCHAPS
Culturally and linguistically appropriate care	Cultural competency and health literacy	Lecture series Health literacy rotation Web-based curriculum	Grand rounds lectures every 2 months, 4 day rotation integrated into a 2 week QI rotation	Grand round speakers, faculty	OSCE: at least 2/year
Professionalism (individual and in the organization)	Organizational psychology	Reflective sessions for residents	One-hour sessions 4/year	Pastoral care expert	Hidden-curriculum survey; Maslach Burnout inventory
Train the trainer	Teaching environment	Faculty development sessions in teaching patient-centered communication	Weekly meetings, monthly facilitated faculty development session Mindfulness exercises, facilitated discussion, review of OSCE-videos	Weekly meetings self-facilitated, monthly meetings with outside expert	Faculty OSCE: at least 2/year, resident evaluation of faculty communication

Curricular Components and Evaluation of the Communication Curriculum. *ECAP*®: English Communication Assessment Profile; *OSCE*: Objective Structured Clinical Examination; for description of surveys please see main body of text

The patient-centered communication program began with a comprehensive baseline skills assessment that provided each participant with metrics and a detailed analysis of oral language competencies. The program then boosted participants’ skills through five 3-h workshops addressing the following areas:

i) Communication strategy (arranging ideas, simplifying complex information, cohesiveness, barriers to understanding)
ii) Vocal image (thought groups, pitch, and emphasis, speed control)
iii) Adjusting language intensity (responding to ideas, framing positions, diplomacy, explaining bad news)
iv) Choosing the right words (avoiding jargon, setting an appropriate tone, changing words for patients and colleagues)
v) Accent improvement (use of rhythm, word stress patterns, phonetics and consonant accuracy).

Each workshop included didactics, videos, and skills practices in a group. Homework after each session was utilized to practice skills.

#### Content and strategy of patient-centered communication

We followed a previously published format for the curricular content in Patient-Centered Communication [15]. This video-aided interactive curriculum included: breaking bad news; developing rapport and relationships with patients; encouraging patients to express what is most important to them; eliciting personal concerns as well as symptoms from the patient; and using active listening, neutral utterances, echoing, open-ended questions, and summary statements during the patient encounter; engaging in social conversation; using appropriate body language, and writing personal narrative/self-reflection to express emotions during challenging situations. Content of the lecture series was repeated and demonstrated in daily work-rounds by teaching faculty.

#### Culturally and linguistically appropriate care

Residents utilized two freely available online resources for this component [16, 17]. In addition, we had a monthly lecture series as well as integrated a 4-day rotation into a 2-week quality improvement rotation. In addition to the web-based curricula, the rotation supervisor reviewed videotaped patient encounters with residents to identify personal strengths and weaknesses and areas for improvement in engaging patients with low health literacy. Residents also assessed the reading level of a patient education pamphlet and rewrote the pamphlet to a more appropriate level. Residents presented the revised pamphlet at the hospital’s Patient-centered Care Improvement Committee at the end of the rotation.

#### Professionalism

A pastoral care expert performed four reflective sessions per year for residents, who invited residents to reflect on ethical dilemmas and their personal experiences with difficult patients or colleagues. These reflective sessions were integrated into an existing ethics lecture series.

#### Faculty development

In addition to direct resident teaching, and because of the importance of role-modeling, we also developed and implemented a faculty development program. For the program, we augmented weekly faculty meetings to discuss teaching and communication challenges as well as team morale. During the weekly meetings, we reviewed faculty OSCE tapes and discussed ways to incorporate communication in teaching rounds. We also arranged for monthly sessions with an outside expert facilitator (certified by the American Academy on Communication in Healthcare) to cover practicing and teaching patient-centered communication. The monthly meetings included brief exercises in mindfulness, free discussion of current challenges and topics, as well as review of the faculty OSCE-tapes to identify and discuss communication strategies and how these could be integrated into daily teaching encounters.

### Program evaluation/instruments

Each component had at least one corresponding assessment instrument. We performed all statistical analyses with SAS 9.2 (SAS Institute Inc., Cary, North Carolina). We tabulated frequency tables and performed a t-test to compare continuous variables. We conducted chi-square analyses to compare categorical variables.

### English communication assessment profile

We measured the linguistic ability with the English Communication Assessment Profile (E-CAP) [18]. We used both speech analysis software and trained rater evaluation to assess residents’ communication. The Web-based testing platform is used to check the resident’s verbal response to a variety of communication tasks [12]. The resident responses were compared with standard benchmarks in more than seventy-five separate areas for indicators of communication competency. The E-CAP measures language ability, communication strategy, information organization, diplomacy, and other factors essential for effective patient interaction. Based on the aggregate benchmark for international professionals, a score of 70 is viewed as the minimum threshold for effectiveness. We assessed the changes in E-CAP score results at the beginning and the end of the one-year training program and analyzed with a paired t-test.

### OSCE

We evaluated the communication skills of residents and faculty with Objective Structured Clinical Examinations (OSCE’s) which provides a valid and objective assessment of communication skills [19]. OSCE’s were held twice per year with two to three communication stations. In addition to evaluating patient-centered communication, four OSCE sessions focused on health literacy (one in 2009, one in 2010, and two in 2011).

The faculty members were invited to participate in the OSCE first and taped to benchmark OSCE performance. Residents had two minutes to read the scenario. Then, they interacted with a standardized patient and were videotaped for six minutes. At the end of the station, the patient actor spoke for one minute into the camera giving feedback for the resident. Each OSCE was later reviewed with the resident by the program director (or, in the case of the health literacy rotation, by the rotation supervisor).

### Calgary-Cambridge observation guide (see Additional file 1)

Two independent raters rated these OSCEs according to a modified Calgary-Cambridge Observation Guide (CCOG) [20, 21]. We identified 21 behaviors from the Observation Guide that we believed could be exhibited in a six-minute patient encounter. The CCOG identifies various communication behaviors (such as “explores patient’s feelings” or “negotiates the agenda”) which are easily observable. Each of these behaviors was rated as “done” (=2 points), “partially done” (=1 point), or “not done” (=0 points). The OSCE score for each resident was calculated by dividing the number of points achieved over the total 42 points possible (=21 behaviors x maximum 2 points), and expressed as a percentage. The resulting OSCE-score represents which percentage of patient-centered communication behaviors from the CCOG the resident used in the encounter.

We compared average orientation OSCEs scores (conducted before exposure to the curriculum) with the final average OSCE scores (after exposure to curriculum) by using a t-test among all participants. We also conducted paired t-test for those residents who had taken OSCE more than once to compared baseline and final OSCE scores. The mixed-effects regression model was used to assess the residents’ performance on OSCE based on three-year curriculum. The average OSCE-score of all participating residents in each year of the program was the dependent variable. We used the following metrics as independent variables: residents’ PGY-level, gender, residency program, medical school location (International medical graduate vs. US medical graduate), and timing of the OSCE (1st versus second half of the academic year). Since some residents had participated in more OSCEs than others (due to scheduling constraints), we also controlled for how many OSCEs a resident had completed. The final model included the variable year (the variable of interest), the timing of the OSCE, PGY-level, and how many OSCEs a resident had completed.

### Maslach burnout inventory (MBI)

Physician empathy is a crucial component of patient-centered communication, but has been shown to decline during residency [22]. We hypothesized that improvements in communication and organizational culture would also be reflected in residents’ professionalism and burnout. Therefore, we measured residents’ degree of professional efficacy, exhaustion, and cynicism by using the Maslach Burnout Inventory (MBI) [23]. The MBI is reported to have internal consistency coefficients of .89, .77, and .74, and reliability estimates of .90, .79, and .71, respectively [24]. The MBI contains 16 questions that are divided into three subscales. The questions, explore personal feelings or attitudes, and participants choose the frequency at which they experience these feelings using a 7-point scale [25]. Before and after scores on the Maslach Burnout Inventory were compared using t-test.

### Hidden curriculum

To evaluate organizational culture, we used a validated hidden curriculum survey, [9] which assesses the degree to which “normal” everyday encounters were patient-centered. This instrument has good reliability as measured by Cronbach’s alpha statistics ranging from 0.67 to 0.93 [26]. The original Communication, Curriculum, and Culture (C^3^) Instrument was developed to explore role modeling, students’ experiences, and support for students’ patient-centered behaviors of faculty, advanced residents, and interns. We reworded those questions to reflect that our subjects were residents (e.g., one item in the original survey is “You hear students telling stories about patients. These stories tend to portray patients as diagnoses rather than unique human beings”. We reworded this question to “you hear other residents telling stories about patients…..”) The survey lists several scenarios and asks respondents how frequently they have experienced various behaviors on a seven-point scale (Always, almost always, more than half the time, about half the time, less than half the time, rarely, never). We compared before and after scores for each group by t-test.

### Faculty development

Faculty teaching skills were assessed through resident surveys. The survey contained six teaching domains; learning climate, communication of goals, patient-centered communication, evaluation, feedback, and self-directed learning. Residents were asked to evaluate faculty on a Likert scale of 1–5 (1 = ‘strongly agree’ being most positive to 5 = ‘strongly disagree’ being more negative.) An average score was calculated for each objective per faculty member in 2010 and again in 2013. Changes in average objective score between 2010 and 2013 were analyzed using t-test.

## Results

Table 2 shows summarized results of our analysis. Table 3 provides demographic data on residents and Table 4 provides demographic data on faculty. Baseline score reflects scores for the academic year of 2009–2010, in which we started to implement our program. Follow-up score represents the scores in the last academic year of the curriculum (2012–2013).

**Table 2:** Performance on OSCE, E-CAP, MBI, and Hidden Curricula

	Baseline score (academic year 2009–2010)			Follow-up score (academic year 2012–2013)			
Test	Mean	SD	95% CI	Mean	SD	95% CI	*p*-value
Overall OSCE	0.70	0.16	0.64–0.77	0.78	0.13	0.73–0.82	< 0.001*
Paired OSCE	0.68	0.17	0.62–0.73	0.81	0.13	0.77–0.85	< 0.0004*
E-CAP	73.4	7.18	71.2–75.6	77.4	5.5	75.5–79.2	< 0.001*
Faculty skills^a^							
Learning Climate	2.07	0.74	1.86–2.28	1.61	0.21	1.55–1.67	< 0.001*
Communication of Goals	2.15	0.71	1.65–2.66	1.67	0.19	1.52–1.81	0.06
Patient-Centered Care	2.08	0.73	1.74–2.42	1.63	0.14	1.56–1.70	0.01
Evaluation	2.14	0.62	1.69–2.58	1.62	0.14	1.51–1.73	0.03
Feedback	2.27	0.75	1.73–2.80	1.72	0.15	1.60–1.83	0.05
Self-Directed Learning	2.26	0.75	1.73–2.80	1.66	0.2	1.51–1.81	0.03
MBI^a^							
Cynicism	2.4	1.55	1.60–3.20	1.77	1.37	1.02–2.53	0.24
Exhaustion	2.59	1.47	1.83–3.34	2.67	1.72	1.71–3.62	0.89
Professionalism	5.13	1.1	4.56–5.69	4.64	1.68	3.71–5.58	0.34
Health Literacy OSCE	0.63	0.14	0.54–0.72	0.72	0.12	0.66–0.77	0.08
Hidden Curriculum	0.60	0.36	0.44–0.76	0.62	0.37	045–0.80	0.89

^a^1 = most positive value, 5 = most negative  *p-value <0.001

**Table 3:** Resident Demographics

Demographic	% or mean (*N*)
Age (years)	32.8 ± 4.9
Gender (male)	36.8 (68)
Ethnicity	
Asian	77.9 (53)
White	17.6 (12)
African-American	2.9 (2)
Other	1.5 (1)
US Trained (Yes)	25 (17)

**Table 4:** Faculty Demographics

Demographic	% or mean (*N*)
Age (years)	35.94 ± 4.81
Gender (male)	50 (5)
Ethnicity	
Asian	80 (8)
White	20 (2)
African-American	0 (0)
Other	0 (0)
US Trained (Yes)	0 (0)

### E-cap

A total of 31 residents completed baseline and follow-up E-CAP tests in 2011 (response rate = 100%). Only incoming residents (15) completed the program in 2012 (response rate = 94%). The average score improved significantly from 73.4% at baseline to 77.4% at follow-up (*p*-value of < 0.001).

### OSCE

Each year, an OSCE was completed at intern orientation, in the 1st half and again in the second half of each academic year, with the first OSCE completed in December 2009 and the latest OSCE completed in December of 2012, for a total of 11 OSCE sessions. A total of 73 residents participated in at least one OSCE (response rate 95%). Of these, 35 residents participated in two OSCEs, 11 residents participated in three OSCEs. Three residents participated in four OSCEs, and one resident participated in five OSCEs during their time in the program. The residents’ average score in 2009 was 70.1%, for 2010 it was 72.7%, for 2011 it was 78.6% and for 2012 it was 77.6% (*p*-value of < 0.001).

**Fig. 1:**
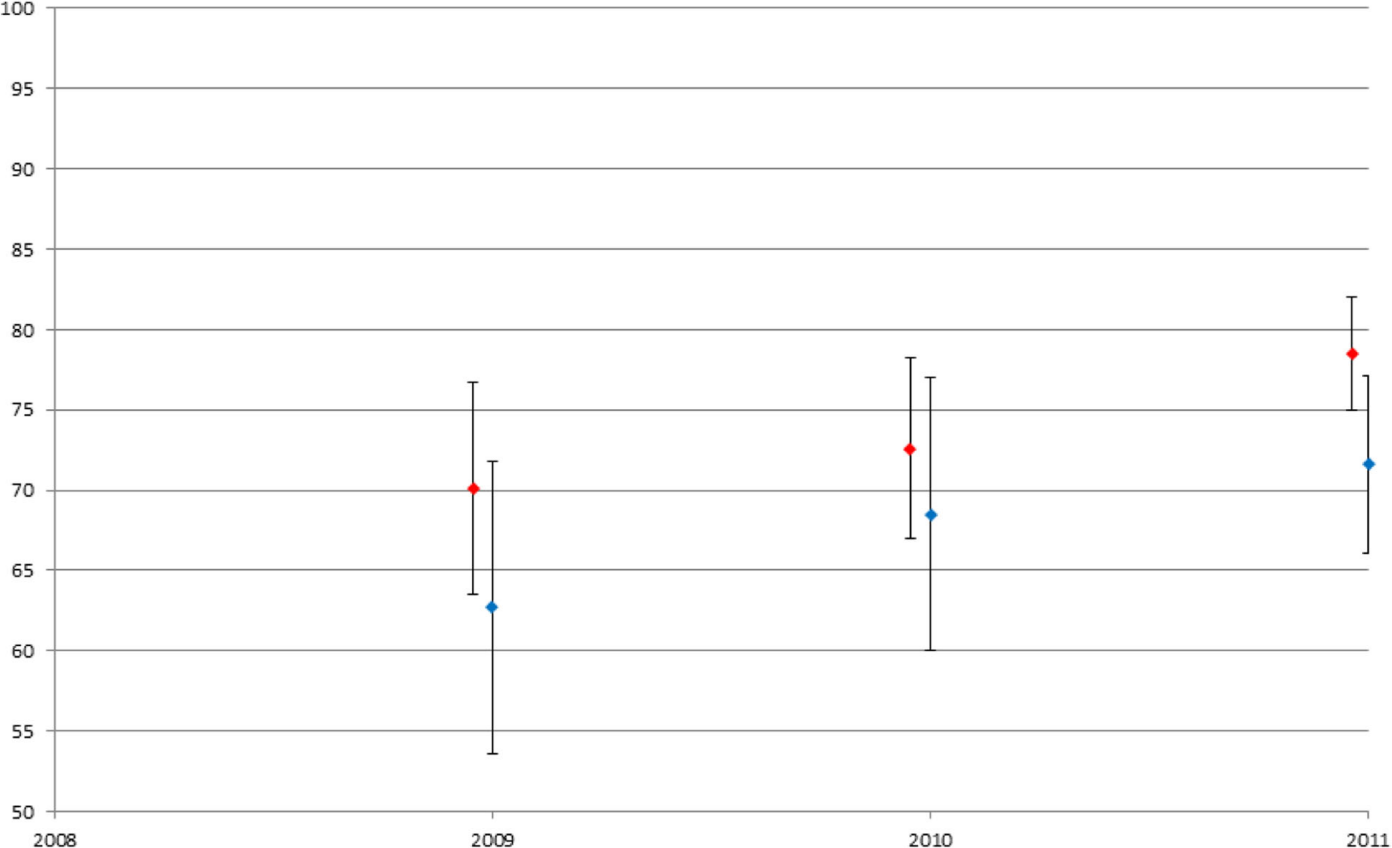
OSCE scores (Overall / Health Literacy)

In paired comparison, average OSCE-scores improved significantly between baseline and follow-up. (0.68 to 0.81, *p* = < 0.0004). As a group, OSCE score progressively improved from baseline to 2nd and 3rd administration of OSCE. For the subset of health literacy OSCE’s, the residents’ scores improved over the three years. In 2009, 12 residents participated with an average score of 62.7%. In 2010, 12 residents participated with an average score of 68.5%. In 2011, 21 residents participated with an average score of 71.6% (*p*-value of 0.08) (see Fig. 1).

### MBI

The MBI survey showed no significant change from the beginning of the program to the end in all three domains, professional efficacy, exhaustion, and cynicism. Exhaustion and Cynicism were high at baseline and remained high throughout the program. Professional efficacy remained at moderate levels throughout the program.

### Faculty teaching skills

In 2010, a total of 17 residents (response rate = 57%) evaluated ten faculty members using the Faculty Teaching Skills Survey. In 2013, 28 residents (response rate = 85%) evaluated nine faculty members using the same survey. Of the 6 teaching domains, learning climate (*p* < 0.001), patient-centered care (*p* = 0.01), evaluation (*p* = 0.03), and self-directed learning (p = 0.03) improved significantly.

### Hidden curriculum

The baseline hidden curriculum survey was administered in 2010 and a follow-up survey in 2013. In 2010, 22 residents completed the survey (response rate = 71%), and the average score was 59.6%. In 2013, 20 residents completed the survey (response rate = 60.6%), and the mean score was 62.2%. This improvement was not statistically significant.

## Discussion

We developed and implemented a multidimensional, three-year curriculum on patient-centered communication to enhance patient-centered care. Out of the six program evaluation measures, OSCE, E-CAP, and Faculty Skills Survey indicated statistically significant improvement. Our approach was similar to the methods used in previously published curricula of patient-centered communication, which include experiential learning, skill-based learning, and fostering of learner self-reflection [27]. However, our curricula included enhanced communication training in linguistics and clear speaking. We are not aware of any other curricula integrating linguistic feedback to residents.

Our residents greatly appreciated receiving direct feedback from the standardized patient. This finding is consistent with previous studies that have found that learners value narrative feedback in OSCEs [26]. Contrary to our expectations, residents’ self-assessed professionalism on the Maslach-Burnout Inventory did not improve. This may have been due to our small sample size. Alternatively, residents’ may have become more self-critical about their professionalism over the course of the program, as residents became more sensitized to their communication behavior. Similar changes have been described in faculty development, where faculty became more and more critical in their self-assessments during faculty development programs [28, 29].

Our study had several strengths. We combined multiple domains of communication with multiple outcome measures that were based on actual communication behavior and implemented a curriculum spanning three years. We used standardized evaluation methods and included the perspective of patients, residents, and faculty. We also assessed and addressed the hidden curriculum of our institution. However, our study should also be interpreted in light of its limitations. As many other curricular studies, we had no control group and used a before and after assessment of changes. We used residents’ ratings for faculty communication, which may differ from the rating of a similarly trained faculty member. Our curriculum required significant faculty time input and was partially funded by a grant. It might not be readily implemented in institutions without such monies. We had to estimate participation rates in some curricular elements. However, we believe that several components could be easily implemented, such as the use of online curricula, taping and reviewing of OSCEs, having residents rate attending physicians’ communication behavior, and feedback from standardized patient-actors. We were not able to obtain individual patient feedback for residents. Further research about the impact of this curriculum on patient outcomes, how lasting the change is, and the impact of physician-patient communication on inter-professional communication are necessary. Lastly, this project only addressed face-to-face interpersonal communication. This study did not explore methods to improve written or e-communication such as emails or video consultations.

### Challenges/lessons learned

Overall, we were able to implement the curriculum as planned. We faced some logistic challenges to ensure participation of most residents with the OSCE’s since we had to account for night shift rotations and vacations. The logistics of securing individual patient assessments of residents were particularly challenging. We were often surprised which components of the curriculum the residents seem to value most, such as presenting their revised pathways at hospital committees, or receiving direct feedback from the standardized patient-actors. Curriculum we have described require significant financial and faculty resources. While this curriculum ended when grant funding expired in 2013, we were able to incorporate components of this curriculum in to general residency training.

## Conclusion

Effective interprofessional learning programs are imperative to promote collaborative practice amongst health care professionals [30]. Observation of performance followed by feedback is considered the optimal method for teaching and assessing professionalism, interpersonal and communication skills [31]. Our study showed that implementing a longitudinal, multifaceted curriculum in a residency program with many foreign graduates is feasible and associated with modest improvement in residents’ actual communication skills. We hope our curriculum can serve as a model for integrating skills-based learning and assessment of observable behavior into other residency programs.

## Additional file

Additional file 1: Revised Calgary-Cambridge Observation Guide. (DOCX 16 kb)

## Abbreviations

ACGME: Accreditation Council for Graduate Medical Education
ANOVA: Analysis of Variance
CCOG: Calgary-Cambridge Observation Guide
E-CAP: English Communication Assessment Profile
MBI: Maslach Burnout Inventory
OSCE: Objective Structured Clinical Examinations
PGY: Post Graduate Year
