# Supplementary material for: Teaching residents to put patients first: creation and evaluation of a comprehensive curriculum in patient-centered communication
Source: BMC Med Educ. 2018 Nov 19;18:266. doi: 10.1186/s12909-018-1371-3 (PMC6245928; doi:10.1186/s12909-018-1371-3)
Supplement: Supplementary file 1 — Revised Calgary-Cambridge Observation Guide. (DOCX 16 kb) [file 12909_2018_1371_MOESM1_ESM.docx]

**Appendix: Revised Calgary-Cambridge Observation Guide**

| **INITIATING THE SESSION** | **Done** | **Partially Done** | **Not done** |
| --- | --- | --- | --- |
| 1. Greets patient | 2 | 1 | 0 |
| 2. Introduces self and role | 2 | 1 | 0 |
| 3. Engages patient by sitting and making eye contact | 2 | 1 | 0 |
| 4. Demonstrates respect | 2 | 1 | 0 |
| 5. Identifies and confirms problem list | 2 | 1 | 0 |
| 6. Negotiates the agenda | 2 | 1 | 0 |
| **GATHERING INFORMATION/EXPLORATION OF PROBLEMS** |  |  |  |
| 7. Explored patient’s feelings about problem | 2 | 1 | 0 |
| 8. Listens attentively | 2 | 1 | 0 |
| 9. Facilitates patient’s responses verbally and non-verbally | 2 | 1 | 0 |
| 10. uses easily understood questions and comments | 2 | 1 | 0 |
| 11. Clarifies patient’s statements | 2 | 1 | 0 |
| **UNDERSTANDING THE PATIENT PERSPECTIVE** |  |  |  |
| 12. Explored beliefs about problem | 2 | 1 | 0 |
| 13. Acknowledges patient’s concerns | 2 | 1 | 0 |
| 14. Encourages expression of emotions | 2 | 1 | 0 |
| 15. Picks up verbal and non-verbal cues | 2 | 1 | 0 |
| **BUILDING RELATIONSHIP** |  |  |  |
| 16. Is not judgmental | 2 | 1 | 0 |
| 17. Shows empathy and support | 2 | 1 | 0 |
| 18. Appears confident | 2 | 1 | 0 |
| **CLOSING THE SESSION** |  |  |  |
| 19. Encourages patient to discuss any additional points | 2 | 1 | 0 |
| 20. Closes interview by summarizing briefly | 2 | 1 | 0 |
| 21. Contract with patient re future goals | 2 | 1 | 0 |

Figure Legends:

♦ Health literacy OSCE scores

♦ Overall OSCE Score
